# Massachusetts Dental Society

**Published:** 1903-11

**Authors:** 


PRESIDENT'S ADDRESS.

*Dr. Andrew J. Flanagan,* Springfield, Mass.—The president's address may be regarded from various points of view. It is rather a common thing for the president's address to paint the dental horizon in glowing colors. In a national organization it is perhaps a grand thing to do that, but it seems to me that in a State society, which is one of the many units which go to make up the great whole, it behooves us to deal directly with that which interests that one unit.

In my address to-day I simply wish to deal with the business of the Massachusetts Dental Society, and with that of no other society.

During the year it has been my pleasure to be associated with various men on various committees, and to become intimately acquainted with their work, and during that year I have jotted down, as is a custom with me, many little things, and I shall speak to-day from the little hints which I have gathered up and which I have thought would be of value and interest to our society.

In the first place, I wish to refer to the word "Membership." Our society needs to grow if it is to be the representative of a profession which is bounded only by the boundary lines of the State. In order to grow it is necessary to have some means to attract membership. It has always seemed to me that no State organization can afford to form itself into a little coterie of members. The State society must pre-eminently represent that State, and in order to do so it must build on long, broad, and familiar lines. You cannot make a select society of it. That is an utter impossibility if you wish to progress.

I speak here to-day with the hope that some means will be devised in the various districts for the coming year whereby we may bring into membership a greater proportion of the legitimate practitioners of this State.

There is a duty devolved on membership. There is a duty devolved on anything if you wish to put your whole power into your work. The old practitioner owes a duty to the young practitioner, and the young practitioner owes a duty to the old practitioner. It is the duty of the old practitioner, when a young man starts in practice in his community, to lend a helping hand; to show him the advantage of being an ethical man and how much better it is to

### MASSACHUSETTS DENTAL SOCIETY.

(Continued from page 710.)

The secretary read the minutes of the last meeting.

*Dr. Maxfield.*—I desire to make a motion, and it is that the president be requested to give his address at the first order of exercises at the afternoon session.

The above motion was regularly seconded.

*The President.*—It has always seemed to me that if there is one society that needs information on the question of courtesy and the question of time, it is a dental society. If you invite gentlemen to come here and read or discuss a paper, the Society ought to be courteous enough to give them precedence when their time has arrived. The president simply waived the privilege this morning of giving his address in order to be courteous. If it is the wish of the assembly that my address should go on at two o'clock, well and good, but I prefer that we should go on with the regular order of business at two o'clock and listen to the address of the president when we have time.

*Dr. Maxfield.* — I entirely disagree with the president with regard to the President's Address. I regard it as a very important matter. I consider the life and prosperity of the Massachusetts Dental Society of great importance, and the constitution plainly says what the president shall do. He shall state the condition of the Society during the term of his office; he shall tell us of its progress or retrogression; and I consider it of great importance, and the only criticism I would make on the programme is that its place is one of the opening exercises. I think it should appear in the programme at a time when we know from past experience when the largest attendance will be.

Dr. Maxwell's motion was then put and carried.

A recess was then taken until two o'clock P.M.

### *Afternoon Session.*

President Flanagan called the meeting to order and resigned the chair to Dr. Waldo E. Boardman, Boston, Chairman of the Executive Committee.

belong to the State organization than to remain outside of its membership. As we grow old it is a pleasure to look back and feel that we have done our share in this direction. It is the duty of the young man, and it should be his pleasure, to recognize the work and the standing of the older men. There is mutual duty in this regard, and it can only hold when it is properly brought to a successful issue.

During the past year this Society has had to appear before the Legislature on several different occasions to further the interests of dental legislation. A committee was appointed last year to take charge of dental legislation. I wish to speak in a plain, unvarnished way to-day, and I do not wish to hurt any one's feelings, but I am convinced that the legislation committee failed to do its work. We had several meetings to further the interests of dental legislation. We had a committee appointed which should have appeared before the Legislature and advanced the interests of this Society, yet when the time came men who were not on that committee, men even who were members of the State Board of Registration of Dentistry, had to appear to further the increase of their own salary. To say the least, it was not courteous, it was not dignified. And the time has arrived when this State Society should see that a small, active, and capable committee is appointed each year to attend to legislative matters. I should recommend that the number of the committee be not over three; that it be made up of experienced, capable men, men who are able to go before the Legislature, to attend committee hearings, and make the wants of this Society known. They certainly should be men of standing and men of reputation. The greatest need of the future is going to come along the lines of legislation. Each year you are sure to see some bills presented that need thorough investigation.

During the last year there was a bill presented to merge the boards of pharmacy, dentistry, and medicine into one board, to be known as the Board of Health. That measure deserved defeat, and it is a pleasure for me to say that we were able to go before the Legislature and defeat that bill in the committee hearing.

There was another bill which was presented from this Society asking for an increase of the salaries of three of the members of the State Board of Registration, so as to put the salary of the members of our board on a par, or an equality, with those of the members of the Boards of Medicine and Pharmacy, and we are pleased to state the bill passed unanimously and was signed by the governor. An amendment to the law was presented last year whereby it was more thoroughly explained how a physician could practise dentistry, and which bound a physician to the observance of certain regulations when called upon for dental services. That amendment was necessary, because if we had proceeded to prosecute under the law as it existed prior thereto we would have been beaten, but the act will now stand the test of law.

There was a bill presented last year to authorize students to practise under the tuition or supervision of registered dentists. The bill was to have been called up at the last session of the Legislature, but was not, and will therefore die a natural death this year.

I come now to the subject of prosecution. Dr. Faxcn, of this Society, spent a good deal of time and thought in putting forth the prosecution committee. It was a grand thought on his part, and it was hoped it would be productive of good results, but we are sorry to say that up to the present time that committee has accomplished practically nothing. The time is ripe and action should be taken during the present year whereby we can have State supervision by the police of the illegal practitioners of dentistry. Dr. Dowsley, the president of our board, has a promise from our present governor that during the coming year he will assist this board in all its work by allowing it the use of the State police. In case that should come to an issue, and the governor should not fulfil that promise, we shall take means to further our end through another channel.

This Society has now arrived at a stage in its growth where two days are not long enough for its sessions. We should have at least three days. It is not fair to the gentlemen who read papers that there is not time for their discussion, and this matter should certainly be taken into consideration at the present meeting, and means taken to extend our annual meetings from two to three days. One of the greatest misfortunes of the profession in a dental society to-day is the lack of thorough discussion of papers; the discussion of a paper is frequently of more value than the paper itself, and by having a three days' session that difficulty will be overcome to a great extent.

The Society has at times been divided on the question of exhibits, but if any gentleman who has been arrayed against exhibitors will step out into the corridor and observe our present exhibit it is safe to say that he will come back converted, for I can truthfully say that the exhibits we have here at the present time are the best exhibits that the Massachusetts Dental Society has ever seen. There is a feeling among the city dentists that exhibits are not necessary. I frequently think that dentists living in the city of Boston, where they have access to the large dental depots and can step in at a moment's notice and secure anything they require, do not appreciate their facilities; it is the country doctor who comes perhaps once a year to our meetings and observes our exhibits who can fully appreciate them, and it behooves us to foster the interests of the country dentists as well as those of our city dentists.

We have had during the year three deaths in this Society, that of Dr. Edward S. Powers, of the Southeastern District, of Dr. J. Searle Hurlbut, of the Valley District, and Dr. Isaac J. Wetherbee, of the Metropolitan District. Dr. Wetherbee was on the honorary list; Dr. Hurlbut was an ex-president,—I believe he was president in 1874. Both of these gentlemen obtained prominence in their profession and did a great deal of work to further the interests of this Society.

The financial condition of this Society at the present time can truthfully be said to be the best in its existence. The treasurer has not on his books any individual who is in arrears more than one year's dues. During the past year various delinquent district societies have paid their bills.

It is a sad thing in a dental society to have much dead wood, commonly called non-paying members, and it behooves this society to cut it off as quickly as possible. If you have members who cannot afford to pay their dues, put them on the corresponding list, but when you have men who can afford to pay, who are good fellows, and who do not pay their way because they will not, you had better cut them off the first year they get in arrears.

There has been a long list of presidents of this Society, and many of our ex-presidents are living to-day. If a man has been honored with the presidency of this organization he certainly owes it to the Society to further its interests after he has left the presidency. When you find in this State organization men who have been honored as presidents who go out and deride the organization, and not only do that but fail to keep up the membership, there is something wrong. That something wrong is not in the Society, it is in the member. The time is ripe to call to account some of our ex-presidents. Some of them have never done a stroke of work for the Society after they have been so honored, and some have even failed to keep up their membership. The stigma attached to a man who has been honored by the presidency and fails to honor the Society afterwards is beyond mention. We should take recognition of these things and point them out in no uncertain manner, so that these men can be called to account.

It is well in electing a president to select one who started at the lower rounds of the ladder; a man who has so started is certainly a stronger man when he reaches the top. He has worked his way. He knows what it means to progress, and is a man who will further the best interests of the Society; and if there could be an unwritten law in the Society that none but those who have worked in the subordinate positions in a thorough manner could gain the presidency it would be of great advantage.

There is a clause in our constitution which fixes the place of meeting as Boston. Now, Boston is a great city. It is commonly called the "Hub," but in order to use a hub we must have spokes, and I think the time is ripe to change that clause in our constitution. It may be wise in the future to have the majority of the meetings in Boston, but we should have the privilege of holding them elsewhere. We have many members outside of Boston, and they should be considered in this respect.

We have a constitution which sadly needs revision; to a great extent it is obsolete.

The Metropolitan District has had many meetings during the last year and a large attendance. The Metropolitan District is in a situation which can easily produce a successful district meeting; it has grown in its membership; its dues are promptly paid; we have no criticism whatever of the Metropolitan District.

The Southeastern District has come forward in a grand manner during the last year. If I remember correctly, it has elected twenty-three members, and that means individual work. The secretary and the treasurer and the executive committee of the Southeastern District certainly did their duty during the past year.

The Central District is still the same old Central District. The city of Worcester alone has eighty registered dentists,—somewhere between eighty and ninety,—and the total membership in that district at present is some thirty-odd members. It is safe to say there are three hundred dentists practising in the county which is taken in by that district, and something should be done to stimulate the work in that region.

The Valley District Dental Society needs no word of praise from me. Its work speaks for itself throughout its entire career, and at the present time in the Valley District ninety-eight per cent. of the ethical men are members of the Society.

The Western District has come to life once more, and we hope it is going to stay there this time. During the last few weeks there was a good deal of discussion in that place as to whether the Western District should disband and become a society to be known as the Berkshire District, or retain its membership in the State Society. After a long debate, participated in by men who have been honored by this State Society, it was unanimously voted to try once more to keep up the membership in our State Society. It is rather a sad thing to find a man who has been honored by the presidency of this organization getting up in a public meeting of dentists and favor leaving the parent organization to form an independent society, and yet that is what occurred in the Western District, but through the efforts of Dr. McLaughlin and Dr. Flynn that district is once more on its feet, and the coming year will tell whether it is on its true feet or not.

No district organization can exist without, first, a thorough secretary, second, a thorough treasurer, and, above all, a very thorough executive committee. When you get those things complete you will have a district society, but not until you do. It is about time the district societies removed from office some of the dead timber which is to be found among their officers. It is a sad thing when men year after year fail to send in a report to our secretary. Such men should never be allowed to remain in office, but should be removed.

We hear at the present time a great deal of the strenuous life. We hear a great deal of the prosperity of dentistry at the present time. Evolution is going on in dentistry as in the world at large, and dentistry is partaking of the prosperity the same as other things. At the present time, if anything is required to be brought to a higher standard, it is that which is covered by the old New England term "manhood." When our district society, our State Society, and national organization attain to that promience wherein manhood is first considered we will have our organizations very near perfection. The greatest need to-day in this organization, the greatest need in the world at large, is that good old principle known as manhood.

Let this Society further its interests and advancement by remembering these things and taking into consideration that the advancement of the organization can only come by the cultivation of principles which go to make up honest and true manhood.

*Dr. Boardman* (in the chair).—I can heartily commend the address of the president in every respect. I know something about that of which he has spoken. The address is now before you for discussion or reference. What is your pleasure?

*Dr. Maxfield.*—I desire to make a motion, but I want to do it with the understanding that I shall not be appointed on the committee. I do not think it exactly fair to a presiding officer, when he makes his annual address in this way and makes so many recommendations, that no particular notice should be taken of it. The president has made some excellent suggestions and something should be done in regard to it. I move that a committee of three be appointed to go over these recommendations and make a report to this meeting before the adjournment, but I make the motion with the understanding that I am not to be on that committee.

The motion was regularly seconded and unanimously carried.

The following committee was appointed by the chair pursuant to the above motion: Drs. L. D. Shepard, George A. Maxfield, and John W. Bailey.

*Dr. L. D. Shepard.*—It seems to me the committee should have more time to consider the President's Address; there is very much in it, and it seems to me the committee could hardly consider all the recommendations and make a report at this meeting.

*Dr. Maxfield.* — I think Dr. Shepard has made an excellent point, and I move that this committee have six months within which to report, and that their report then be sent to each district society.

The motion was regularly seconded and unanimously carried.

President Flanagan resumed the chair.

*The President.*—At the National Dental Association meeting at Niagara last year there was presented a paper of great worth and interest, entitled " A Bird's-Eye View of Pathology and Therapeutics," by a gentleman who is associated with a college in the city of Nashville, Tenn., and one who is well able to come before this organization to-day and read an interesting paper. I have great pleasure in introducing to you D. Rankin Stubblefield, A.M., M.D., D.D.S., of Nashville, Tenn.

*Dr. Stubblefield.*—Mr. President and gentlemen of the association, it would be an injustice to my feelings to say that I fail to appreciate the compliment and the pleasure of being with you. I have in all my professional years known of this contingent of the professional body; in fact, we of the West or Middle West look, as it were, to the rising sun for inspiration, for tradition, for all that is known or to be known.

Dr. Stubblefield then read a paper entitled "Sanitalogy: A Discussion of the Health Principle."

(For Dr. Stubblefield's paper, see page 769.)

*The President.*—We expected Dr. Andrews to open the discussion, and in his absence I will ask the secretary to read a letter received from him.

"1044 Massachusetts Avenue,
"Cambridge, Mass.

"My dear Dr. Boardman,—I regret very much that I shall not be able to be at the meeting to discuss that paper. It is an excellent paper, and I agree with what he says. I could only express my pleasure. I am going to try and get it on Thursday.

"Very truly yours,
"R. R. Andrews.

"June 1, 1903."

*The President.*—In the absence of Dr. Hopkins I will ask Dr. Stockwell to open the discussion. (Dr. Stockwell did not respond.)

*Dr. Shepard.*—I have listened with great pleasure to the paper and paid very close attention to it, and have been very much interested in it. I hardly feel competent to discuss it in the way which it merits. In connection with the investigations of Dr. Black and others, there have been statements made that will hardly harmonize with the experience of years of practice. When we are told that there is no difference in teeth in their resistive qualities, we have to say that in our experience it has not been so. When we are told by the investigators that teeth do not change in their composition from the time they are erupted until old age, we feel that science does not speak the entire truth. We know from clinical experience that teeth do change internally from youth to old age, whatever scientific investigation may affirm. Every practitioner of experience has adopted a method of practice based upon his faith and in opposition to these statements. Who to-day would presume to treat diseased teeth of a child of twelve or fifteen as they would the same teeth at forty? And why? The teeth are not the same in composition. I need not enlarge on that; you all know what I mean.

What is the excuse for the myriad of dressing fillings, if you will call them so, as some have, which are inserted in the time of adolescence, if the teeth have the same resisting power to attack as they have later?

I have, as the result of experience, more faith in this principle which we have listened to of the inherent tendency implanted in our organization tending to health and recuperation.

*Dr. C. F. Stockwell* (Springfield, Mass.).—When the president called upon me a moment ago I hesitated to speak because of two facts,—first, I was not able, on account of my infirmity, to hear it all; and further, because it seems to me this is one of those papers which, in order to discuss intelligently, one needs to have—well, a good Sunday afternoon out of doors to study it and think about it in its various aspects.

In so far as I was able to catch the drift of the paper, I certainly do agree with everything the essayist said. Furthermore, as he read his paper I could scarcely help feeling that this paper, with some others we have heard recently, marks an epoch in dental science. It is an indication of what I believe to be, in effect, the turning of the tide which has been so prominent in the last six or eight years, in the trend of which Dr. Black and others, as Dr. Shepard has said, have been the chief leaders.

Again, several times during the reading of the paper a scientific axiom came to my mind, which, in short, is that every individual is the product of three principles, if we may divide these principles by strict and definite lines; they are substantially one, but with three different aspects; that is, every individual is the product, first, of heredity, second, of environment, and third, of an innate tendency to differentiate. Some years ago it was generally held that the most dominant of these was heredity, and that what man, or any born of organic life, might be as an individual was due to a larger extent to heredity than to either of the other two principles; later on the emphasis came to be applied to the second of the trio, that of environment. . . .

At the present time, however, if I understand the drift of scientific thought, the emphasis, by the most penetrating minds, the most advanced minds in the scientific world, is beginning to be placed on the third principle,—the innate tendency to differentiate. This is, practically, the same principle which the essayist has dwelt upon and emphasizes so strongly and ably; it is what he called the "health principle," which, if I were to name it myself, I should perhaps call the life principle or the principle of life. It seems to me that he is in accord with the present most advanced biological, physical, or chemico-physical views of the scientific world as I understand them. These views, at least, it seems to me, greatly strengthen the essayist's argument.

Many of our best biologists and physiologists the world over hold to-day that life or the life principle precedes organism; that, in fact, it builds its own structure or body. If that is true, if life principle precedes organism, why, of course, it is the principal fact for us to deal with in medical science and dental science as well; and we might go further and say we must not forget that principle in even social science, to say nothing of philosophy in general.

I believe the essayist is right, and, as I said before, I believe this paper will mark an epoch. There is reaction against the idea that environment is the one all-essential principle, or that heredity tells the whole story. We must recognize the fact that Life—the health principle, as the doctor calls it—is the essential factor, the bottom fact underlying all other facts.

If I could have, as I said a moment ago, a day with the paper quietly by myself, I am satisfied I should find many things in it I would like to talk about; but I hesitate about saying all that might be said now and here. In any event, we owe the doctor a large debt of gratitude for coming so far, and for giving us a paper so interesting and suggestive.

*Dr. Stubblefield* (closing the discussion).—I thank you, Mr. President and gentlemen, for the tolerance which you have exhibited, the patience, that virtue which is so to be commended.

I realize that it was handling explosives, and certainly hot things, to take a subject that seemed to be so foreign to our specialty and to stay so long on it. I did flatter myself that I had started something that would at least not be misunderstood, and the very first man who opened the discussion showed that he had misunderstood. But he, after a while, came around and admitted that I was not as bad as I looked; therefore I congratulate myself. "All's well that ends well."

It is not necessary for me to bring up all that he said, but it was a clear misunderstanding. I did not say and I do not intend to say, and I do not want to be put on record falsely,—although I am a very great believer in standing like a man for my convictions,—that I said or meant to say that there was no change in the structure of the tooth from youth to manhood, that those changes in the teeth do not go on. No. What I said, brought into plain, every-day, common-sense language, was, that there was a principle that had not been fully recognized. We have been standing on the outside of the organism punching at it, and we did not, at least consciously and intelligently, recognize there was something inside of the man that had a power of individualizing. Dr. Stockwell has very properly paraphrased it as the effort of the organism to make itself—to make its own environment, so to speak.

I thank you very much for having permitted me to read such a long paper. I wish you would regard it as something that can be looked upon as an every-day, common-sense thing, and as talking about something that we have demonstrated. I don't care what you call it, but it is like the practice of medicine. I think we are over-awed too much, that the doctor has assumed that he must puff himself up, take his gold-headed cane and his high hat, and walk over us. The doctor, after all, is nothing but a man, an educated man, to be sure, but he has his limitations, as any other man, and we can get close to him and get the good out of him as from any other man. So with this subject. This is an effort to take a common-sense, every-day, homely view of or hold upon a principle that possibly has never been thoroughly recognized. I do not think, if there had been a doctor here, while he might have bristled in the beginning, that he would have objected in the end to what I said. He knows there is a common-sense view of the practice of medicine, and that the world at large does not recognize it. It is the common-sense view of a principle underlying teeth and every other part of the living organism that I was trying to slip into you, in a dental association, and then make my apologies afterwards.

I am very much indebted to you.

*The President.*—Some years ago there came to the city of Springfield a dentist to do some porcelain work, and there gathered around his chair at that time, as he inserted a porcelain filling in the mouth of my assistant, several dentists, and for the last two years every time that young man goes into the office of these aweinspired dentists he is invited into the chair and they take an excavator and start to pull out half the lateral tooth, wherein is inserted an excellent porcelain filling put in by Dr. Patten, and up to the present time they have not been able to pull that filling out. I have great pleasure in introducing to you Dr. Charles C. Patten as the next essayist.

Dr. Patten then read a paper entitled "The Porcelain Art in Dentistry."

(For Dr. Patten's paper, see page 825.)

*The President.*—The next paper is one by Dr. James Ross, of Fitchburg, entitled "Porcelain: A Fad or a Fact."

(For Dr. Ross's paper, see page 847.)

DISCUSSION.

*Robert T. Moffat, D.M.D., Boston.*—The title of Dr. Patten's paper led me to think that he would mention something besides porcelain fillings, and I expected to bring up one or two points with reference thereto, but I find that he has limited his paper to porcelain fillings, and therefore I shall not have very much to say.

Among the various difficulties which we have in porcelain Dr. Patten did not mention the question of lights and shadows, which is one of the many difficulties which beset the worker in porcelain and which should be well considered. I am sorry there is not a black-board here, so that I might make my meaning clearer in reference to that special point. It goes without saying that in restoring the contour of a tooth with a porcelain filling such contour should be restored exactly. Taking a central incisor with an approximal cavity occupying about one-third of the area of the visible surface, if the labial contour of the filling is not exactly continuous with the labial surface of the remaining tooth you will get a different appearance at different points of view. In other words, if you stand directly in front of such a tooth it may probably look all right, but on moving to one side or the other it may appear darker or lighter according to the direction from which the light strikes and reflects. In inlays particularly the question of shadow changes a great deal with the depth of the filling. In a shallow filling the cement will show through, and if the inlay is only a little deeper than the natural enamel of the tooth you will get considerable apparent change in color when the filling is set with cement. An allowance should be made for that in matching the porcelain, and it should be made about a shade or half a shade lighter than it is expected to be when set.

The question of the color of the cement used in setting the inlay or filling affecting the ultimate shade is worth considering. Ordinarily I use the white Harvard cement; there doubtless are others just as good, but this is what I use, and find it answers every purpose excepting in a yellowish tooth, in which case I use what is known as No. 3 color, or yellowish white.

Some dentists say that these fillings are not practical. These men have perhaps had little or no experience with them and do not care to go on with the work, it taking too much time and they feeling it does not pay them from that point of view. I advise you all not to be one of these. Porcelain has its uses and should be utilized, not everywhere, but in proper cases. The fees should be made commensurate with the amount of skill required in the work. No man should say it does not pay, provided he can properly do it. When one starts on porcelain work, if he starts with a small filling he naturally works up to the larger and more difficult ones, especially the approximal cavities, which I think are the hardest to do. But they need not finish there; when they have gotten to a stage where they can make all kinds of porcelain fillings they have not reached the limit of the capabilities of porcelain. Crowns can be carved from porcelain, but not with the same preparations used for filling. Porcelain for carved teeth and crowns is a different material. Crowns may be made from these so-called porcelain enamels, but they are not very strong unless made under pressure, —that is, in a mould. The porcelain used for a carved crown is different, but it is nothing new. I expected to discuss this subject, but Dr. Patten did not mention it in his paper.

With regard to the appearance of porcelain as compared with gold in the mouths of professional people, such as actresses and public speakers, it has been my lot to have a few such as my patients. I recall particularly the case of a young woman who had rather good-looking teeth and very good gold fillings in the front teeth, but they were very conspicuous when she was on the stage, and from certain parts of the auditorium they looked black; they were very good fillings, but they looked like holes in her teeth, as she expressed it to me, and I found all that difficulty was removed by the use of porcelain fillings. It was a matter of dollars and cents to her, and made quite a difference in her engagements. The same thing occurred with an actor who had some gold caps on his teeth, which were removed and replaced by porcelain, which made a great improvement.

A patient sometimes, after having had two or three porcelain fillings in visible cavities, likes them so well as to want them everywhere. That is something you must guard against, and the patient must be given to understand that they are not suitable for every case.

It is surprising to see how much longer porcelain fillings will wear than some other fillings in the same mouth. I recall particularly an elderly patient who could not stand much work being done on her teeth at one time, and it was necessary to use much cement and gutta-percha. In September, 1895, I put a porcelain filling in this woman's mouth, and she is using it yet, while in the adjoining tooth she had had a gutta-percha filling replaced at least four times, and I do not know but more, in that length of time, showing the difference in the wear of different fillings in the same mouth. There has been no washing out of the cement and no change so far as I am able to observe in the condition of the porcelain.

Dr. Ross stated that porcelain has come to stay, and I certainly think it has. It is looked upon by many, especially by the younger practitioners, as a comparatively new filling, but it is not. In 1897 Dr. Jenkins showed the porcelains in this country, and since then there has been a great reawakening of interest concerning them, but they are certainly a great deal older than that. I have records of fillings that were put in by Dr. A. H. Parker, of Boston, and also some by my father away back in the early eighties, and I know that some of those fillings are present to-day in the patient's teeth, and have never come out. I saw a porcelain filling within a month which has been in just thirteen years; it was put in by Dr. Chamberlain, of Rome; it was not made in the manner in which we make them to-day with a matrix, but from a piece of porcelain taken from a porcelain crown, which was chipped off and ground to fit the cavity fairly well. We would not consider it a good fit to-day, but in that day it was good enough, and that was on the cutting edge of an upper right cuspid, where it was subjected to a great deal of wear, and it has never been out during all that time. That shows they will stand for thirteen years at least.

Dr. Ross spoke of not using a dam on account of the change of color. That is a point worth considering. When you come to set the filling, if you show the patient the filling before drying the tooth and setting it, the patient will be quite pleased with the result, but when you have dried your tooth preparatory to setting it there will be a decided change, and that is very apt to be disappointing. If the color is properly treated in the beginning, that is, made a little lighter than the tooth in question so as to make allowance for the cement, that color will change within two or three days, so that it will be greatly improved, and if the patient is discouraged in the outset you can hold out the comfort that it will look better in the future.

The question of stained joints comes up sometimes, and I think there is but one reason for the cause of that. In these modern low-fusing bodies, if you do not take the greatest care in getting a perfectly smooth and polished edge for the filling, you will get that discoloration owing to the little saw-like edge on the filling, which will catch and hold dirt. I have noticed that where the fillings have been absolutely smooth there has not been such trouble.

The porcelain fillings of the present modern material should be etched with hydrofluoric acid on the cavity side previous to setting, and unless that is done the filling will be likely to come out sooner or later. These modern porcelains are practically a solution of silex in glass, and unless the glass is roughened the cement will not get a strong and firm hold.

*Walter I. Brigham, D.D.S.,* South Framingham.—For the last two years it seems as if no convention would be complete if it did not have one or more papers or demonstrations on porcelain inlay. Many men have given us the benefit of their time and experience in demonstrating the work, and Dr. Ross and Dr. Patten have always been very liberal in giving us their time and explaining their methods.

There is no use denying that porcelain has come to stay and that it does fill a place in dentistry which nothing else has ever done; but if a man tries to use it everywhere and anywhere before he fully understands its use, he will have any number of failures. Many men come here and talk of their successes, but few of them speak of their failures. I have put in a great many porcelain fillings, many of which are giving good service to-day, but I have had some come out. That, however, was no fault of the porcelain, but rather my lack of ability or failure of observation in doing the work.

Some men, especially Dr. Reeves, of Chicago, advise the use of porcelain without any undercutting, practically without any cavity, and sticking them on to the side of the tooth. He may be able to do it, but I cannot. I do not put in any inlays now unless they enter the tooth quite a distance, so as to have proper retention, and if it is possible all inlays that I put in will only enter the cavity from one surface, from one way, and, in order to be dislodged, would have to come out that way.

Some men look at the use of porcelain in a peculiar manner; that is, they look at it with distrust and very much with the feeling that they really hope it will not succeed. They like to see a man have his failures. I cannot attribute this to anything other than the fact that perhaps they are not willing to try and do the work that is necessary to perfect one's self in its use. But we who graduated long ago must work along with porcelain and acquire skill. If we do not, lo! we will be a back number, for the student who graduates to-day is going to know something about porcelain, and he will surely use it.

Dentists are looking for things that are easy to do. Only a few years ago archite cement came into use; it was to be a universal filling, and every dentist in the Union, nearly, bought a package, and yet you could hardly induce some men to attempt to use porcelain, which is as far ahead of archite cement as one could imagine.

If you will find out the cause of your failures and eliminate that factor, you will soon gain ground and the failures you once had will all pass away.

The realm of usefulness of this material is almost unlimited in the hands of some, while with others its limits may be much circumscribed. But it is only by knowing its usefulness and your own ability to use it and then by constantly extending them that you can get the best results.

I do not think that the difference in the kinds of porcelain amounts to very much. All of those in use to-day are good. Some men claim that with one you cannot do what you can with another. A dentist told me that with the Jenkins body I could not do a certain thing, that it was an impossibility; I told him I thought

I could, and I tried it and did it, as any man could if he would use the care necessary and become familiar with the peculiarities of that certain porcelain.

It is hard to speak much of the preparation of cavities for the reception of porcelain fillings, but to my mind here lies the fundamental principle of success. As I said before, I now make no porcelain fillings that do not enter the teeth for quite a distance, and if it is possible I have the walls as nearly parallel as can be; then, with undercuts, I get quite a strong hold, and in some cases have them practically lock themselves in.

Dr. Patten spoke of the use of platinum with the Jenkins body. I do not doubt that Dr. Patten has had excellent success with that, but I never had; I can do far better with the Jenkins body with gold than I can with platinum. The time of investment is *nil* compared with the rapidity with which you can work and the assurance of success. With the gold for a matrix, and invested, I am sure my result is going to be just as I expected, but my experience with platinum has been that the Jenkins body, although a low-fusing body, adheres more strongly to platinum than any of the other bodies, and as it shrinks it has a tendency to draw the platinum out of shape, especially if it is a large inlay such as is used for a bicuspid or a molar; with the smaller inlays you may not have that trouble.

Speaking of its use in bicuspids or molars, I think those are ideal places for this filling; you can build up on the approximal cavities extremely well. I have seen several bicuspid fillings which took in the coronal, mesial, and distal surfaces, where there was but little on the buccal and palatal walls standing, and with the porcelain in there the tooth was restored to its normal contour and the gum covered it nearly one-eighth of an inch.

In children's teeth we use cement for certain reasons, and in these cases to my mind a porcelain filling is an ideal filling, because you can get the benefit of the cement next to the tooth-substance, and you can get the durability of the porcelain; in the majority of cavities the filling is such that you can put in a porcelain filling that will last for a good many years.

One hardly knows what to call a success in a porcelain filling; if it should fail after several years, it is to my mind a success, as all things that we as dentists do are liable to fail, and in aged people it does fill the place perfectly. What can you do with incisors or cuspids? You may put in a gold filling, but it would be a large one and far above the enamel line, at the cervical wall, but you can restore them with porcelain with a great deal of success.

There will always be just the same difficulty in different men's inlay work as there is in different men's metal fillings of gold or amalgam. There will be some men who will put in inlays that will work beautifully and give no trouble and last for years and years, while other men will have failures.

The preserving qualities of porcelain are unequalled, and that is due in a great measure to the cement, in the conditions in which it is used. I have great faith in the antiseptic qualities of the cement as it is closely held under the inlay. A cement filling has antiseptic qualities which are soon lost in an open filling in the mouth, but which are retained for a long time under a porcelain inlay, for no matter if that does wash out in the joints you will never see decay. Either bacteria will not live against the porcelain, or else there are antiseptic qualities in the cement which prevent decay.

There is one thing against porcelain to us as dentists, and that is that the filling is an entirety, and when it comes out it comes out as a whole, and the patient is very likely to feel that it is our fault. If you put in a gold filling which has remained in for three or four years and then decay commenced around it, the patient would not question it, but if the filling comes out intact the patient questions the value of it.

I was very much pleased with Dr. Ross's paper and with all he said concerning a dentist as a useful man,—his usefulness and his willingness in a way to be a philanthropist; that is, to do the best he can under all circumstances; and if a man can use porcelain and thus be of greater service to his patient, then it is porcelain he should use. But the dentist should strive in every way, as every other man should, to do the very best he can. It is only by serving your patient well that you can succeed in your profession.

There seems to be a great difficulty in gauging the color in some teeth, but it seems that the most normal colors are the easiest to match, the hard ones seem to be the abnormal ones.

The kinds of cavities in which porcelain can be used seem to be almost unlimited; I use it a great deal in building up molars and bicuspids instead of using gold caps; I do not think I put on ten gold caps in a year, and my hands are never idle.

We will sometimes have trouble in the shading of the porcelain, but to my mind a great deal of that is due to the translucency of the porcelain, and people have tried to get a translucent porcelain, which they should not have; they should have an opaque body with a glassy surface, which gives them a translucent surface and then the cements do not change the color much; that is overcome to a great extent by using the modified bodies beneath and coming to the surface with the lighter ones; you then get more of an opaque body and a substance which the cements will not change the color of.

PROCEEDINGS AT THE BANQUET HELD AT THE HOTEL LENOX, WEDNESDAY, JUNE 3.

*President Flanagan.*—I thought, as I heard a few moments ago the quartette singing "Weep no more, my lady," they were going to say that they would weep no more for the toast-master, and I was glad to hear they were weeping for some one else. (Laughter.)

In this Society there is a committee known as the Committee on Banquet and Entertainment. That committee has had a great service to perform in the last few years for this Society. I believe last year the committee was made up of identically the same members as it is this year, and on it are Drs. J. F. Dowsley, George C. Mitchell, T. J. Barrett, and Eugene H. Smith; and as I sat here this evening I thought why is it they desert me? Last year they spread themselves around the head of this table surrounding their toast-master to brace him up and give him all the encouragement possible, and here to-night the only man I find true to the last is my good friend Dr. E. H. Smith; the rest have deserted me.

Coming here to this the thirty-ninth annual meeting of our good Massachusetts Society, I wish to extend, in behalf of this Society, a cordial greeting, a greeting that comes from the heart and a greeting that may continue year after year until our good Massachusetts Society has attained that zenith which we are all striving for,—perfection.

In our society there is much that is serious, and for once it is well for us dentists to gather around the festive board and come down to the more social and the better part of life, and on behalf of the members I extend, especially to the ladies, our greetings and our thanks for their attendance here to-night.

My thoughts stray to a city not more than a few hundred miles away which has always been foremost in many educational events, and which to us practitioners of dentistry appeals more closely because of its associations. I can remember my two years spent in that beautiful city, when such men as Garretson, White, old Dr. McQuillan were there, and where there are at present such men as Dr. Kirk and our good friend who graced this board last year, Dr. Truman; and I am proud to say that we have here to-night, to well represent that city in all its best interests from the stand-point of dentistry, that noble gentleman who graces the chair of Operative Dentistry and Dental Histology in the University of Pennsylvania, Professor Edwin T. Darby.

DENTISTRY.

*Edwin T. Darby, M.D., D.D.S.*—MR. TOAST-MASTER, LADIES, AND COMRADES IN DENTAL SERVICE,—When I received a very courteous invitation to be present with you to-night I felt that I could not accept it, and yet I had not the courage to decline. We were in the midst of our examinations at home, and I knew that if I accepted this invitation I must do my work before leaving or I could not come. But a similar invitation had been extended to me so frequently in the past that I felt I should be ungrateful and might be considered discourteous if I did not accept this time. Besides that, I had a desire to meet the gentlemen of the Massachusetts Dental Society. I have known a few of you for many years, and I have known some of you for a less number of years, but I have known of your worth for a long time.

The men of New England have always impressed me as self-respecting men. There is a good deal in that word "self-respecting." I remember when I was quite a lad I went to Philadelphia to study dentistry. I went into a boarding-house, and there sat at the table with me a dear old Quakeress; she turned to me one day and said, "Thou art from New England, art thou not?" and I said, "Not exactly, madam; why do you ask the question?" She said, "Thy speech betrays thee." (Applause.) I went into the college, and the students called me a Yankee. But I said, "No, I am not a Yankee, I came from New York State, but I come of a Yankee ancestry; my parents came from New England." The nearest approach I can come to a Yankee or a New Englander at the present time is that I am a member of the New England Society of Pennsylvania, and am proud to be a member of that body. (Applause.)

I observe that you have me announced to-night to speak upon the subject of Dentistry. Some one has said that in order to make a good after-dinner speech the speaker ought to know absolutely nothing about his subject, or else he should know everything about it. I have sometimes heard after-dinner speeches (and you may hear one to-night) where I thought the speaker knew absolutely nothing about the subject, and I have heard other speakers who ought to know everything about the subject make perfect failures.

Pardon me if I digress just a little. Fifteen years ago I moved out into the country five miles from Philadelphia, thinking perhaps it would be better for my health and that of my children. I found that I had located in a community of Friends, the Society of Friends, or Quakers,—excellent people they are too, and I have found them so after fifteen years. I had not been long among them before they worked me into service. I do not know just why they did it, but they made me president of the Building Association. They made me president of the Country Club. They made me president of the Sewer Corporation. They made me a director in the bank. They made me president of the Council, and they then made me chief burgess of the borough, which position I hold at the present time. But that is not all they did. They were going to work me still more, and they asked me to lecture. In some way they found I had been in the far East, in the Orient, and they asked me if I would not deliver a lecture on that subject. You know these Friends, these Quakers, are always on the lookout for every bit of information they can get. Then they asked me if I would not deliver a lecture on Egypt. It was some years since I had visited Egypt, and it made me some work, but I said, "Yes," and gave the best I could as a lecture on Egypt. Then they learned I had visited Yellowstone Park, and asked me if I would not deliver a lecture on the Yellowstone Park, and I said, "Yes," and prepared a lecture on that subject. Then the next year they came to me and said, "We want you to give a lecture on Temperance." (Laughter.) I said to the chairman of the committee, "Now, I am not the man to lecture on temperance, because I am just a little bit of a tippler; I occasionally take a glass of wine if I want it, and I think a man to lecture on temperance ought either to be a total abstainer or a confirmed drunkard." (Laughter.) I said, "Let me tell you a story, my dear fellow, and it will illustrate just what I mean. A few years ago there was to be a very important trial in the courts involving a good deal of money. Two men were subpœnaed to testify on behalf of one of the parties to this suit. These gentlemen were both of the name of Wood (mark the name,—Wood). They were both elderly gentlemen; in fact, they were very old, but well preserved. The first brother went on the stand and gave his testimony in a very clear, concise, comprehensive, and satisfactory manner. When he was about leaving the stand the judge said to him, 'Mr. Wood, I have been very much interested in your testimony, and I feel impelled to ask a few questions. You seem to be a man well along in years; would you mind telling me your age?' He said, 'I am ninety-one years old, sir.' The judge said, 'Well, you must have had wonderfully good habits to be so well preserved after all these years; have you ever been in the habit of using tobacco?' 'Never touched it in my life.' 'Have you been in the habit of taking intoxicating liquors?' 'Never tasted it in my life.' 'Have you kept good hours?' 'I have been going to bed about eight o'clock at night for the last thirty or forty years.' And then the judge turned to the members of the court and said, 'You see the importance of living a good, temperate, and orderly life. I am very much obliged to you, Mr. Wood, for the information you have given.' The witness took his seat and the other brother went to the stand and gave testimony as clear, concise, and satisfactory as his brother had done. When he was through the judge said to him, 'I beg your pardon, sir, but you are a brother of the gentleman who has just testified?' He said, 'I am.' The judge said, 'Will you tell me your age?' He replied, 'I am ninety-three years old, two years older than my brother.' 'Would you mind telling me what your habits have been? I suppose they have been like your brother's, or you would not have outlived him and been so well preserved?' 'On the contrary,' he replied, 'I have not been to bed sober in fifty years; I have used tobacco in every form since I was a child; and as to going to bed, I have not retired until twelve or one o'clock in thirty years.' (Laughter.) 'Well,' said the judge, 'I have always heard that Wood to be good must be either very wet or very dry.'" (Loud laughter.)

Now you see, ladies and gentlemen, what I mean by saying that in order to make an after-dinner speaker one must either be very ignorant of the subject on which he is about to speak or very well informed.

The supposition would be that I ought to know something about dentistry, but I declare I am a little at a loss to know just what you want me to say about dentistry. If you want me to say, as some men have said, that I am ashamed of dentistry, I am not the man to say it, because I have always been proud of my profession. I have always been ready to extol my calling, and when I see a man who is ashamed of his profession I feel ashamed of him, and I do not think we have any occasion to be ashamed of dentistry. When I recall the founders of American dentistry I find they were gentlemen who ranked equal with any and all professions fifty, sixty, or seventy years ago. When I recall such men as Horace H. Hayden, Chapin A. Harris, Edward Hudson, Eleazer and Jehiel Parmley, Solyman Brown, Amos Wescott, John Reed, John Allen, and a hundred men that you could recall, or that I might if time permitted mention, I am not ashamed of the founders of American dentistry. (Applause.) Has any profession within our knowledge made such strides during the past fifty years? Has any profession made such strides during the past twenty, or even the past ten years, as the dental profession? Is there any profession in which the work is more positive, more demonstrable, than in ours? Look, for instance, at the changes which have been made and the improvements that have occurred within the past forty years. I can recollect when there were but three dental schools in America; to-day there are fifty-eight. I can recollect when the curriculum of dental colleges was two years of four months each year. What is it to-day? A course prolonged to three years, and now about to be extended to four years, equal to that of medicine in our best schools. If the medical profession spends but four years acquiring knowledge to practise all of the specialties of medicine, and yet we are to devote four years to the study of a single branch of the healing art, then the dental profession has made a greater advance, perhaps, than any other.

Then what about our service to the community? When you take the aggregate of human suffering that is every year prevented, when you take into consideration the pain that would have been suffered from exposed pulps had you not arrested the decay before it reached that pulp, when you take into consideration the comfort that you have given the community in preserving their teeth during life, when you take into consideration the comfort and health of the patients you have benefited by the restoration with artificial dentures of the organs which have been lost, can you be ashamed of the work that you are honestly doing and of the profession which you represent?

I say, as I said a few moments ago, I am proud of dentistry and of the wonderful progress which has been made in the dental profession. (Applause.)

Furthermore, I think the community at large is appreciative of the services which it receives at our hands. It has sometimes been said that the dental profession does not take a positive social stand in the community. I tell you, and you know from your own experience, that no dentist can attend to social functions and attend to his dental practice at the same time. His work is so exacting, the demands upon his physical and his mental powers are so great, that he could not if he tried attend to both of these and the work in his office.

We are doing in the dental profession just what is being done in the other professions. We are doing our best. Our most generous efforts are being put forth for the good of mankind. My brother here to the left (Rev. Dr. Moxom) is doing what he can to elevate the souls of men out of the darkness into God's effulgent light; the medical men are doing what they can to prevent human suffering, to alleviate the miseries of mankind, and to make the world healthier and better. We, gentlemen, are doing just as grand a service. We are preventing human suffering. We are alleviating the suffering of our kind. We are doing the best we can for the world according to our enlightenment.

"All are architects of Fate,  
Working in these walls of Time;  
Some with massive deeds and great,  
Some with ornaments of rhyme.

"Nothing useless is, or low;  
Each thing in its place is best;  
And what seems but idle show  
Strengthens and supports the rest.

"For the structure that we raise,
Time is with materials filled;
Our to-days and yesterdays
Are the blocks with which we build.

"Truly shape and fashion these;
Leave no yawning gaps between;
Think not, because no man sees,
Such things will remain unseen.

"In the elder days of Art,
Builders wrought with greatest care
Each minute and unseen part;
For the gods see everywhere.

"Let us do our work as well,
Both the unseen and the seen;
Make the house, where gods may dwell,
Beautiful, entire, and clean.

"Else our lives are incomplete,
Standing in these walls of Time,
Broken stairways, where the feet
Stumble as they seek to climb.

"Build to-day, then, strong and sure,
With a firm and ample base;
And ascending and secure
Shall to-morrow find its place.

"Thus alone can we attain
To those turrets, where the eye
Sees the world as one vast plain,
And one boundless reach of sky."

*President Flanagan.*—We have in the community of Springfield a gentlemen who is greatly interested in historical investigation, a man who has responded to many invitations throughout the United States, along certain well-known educational lines, and in introducing the speaker this evening I wish to emphatically say that "The Main End" of the Rev. Dr. Moxom's career in Springfield has been for the uplifting on the broadest and best terms of the people of that community. I have great pleasure in introducing Dr. Moxom.

*Rev. Philip S. Moxom, D.D.*—Mr. President, Ladies, and Gentlemen,—We are often thankful to get to the end, even of a banquet. I do not know how many years ago it was, but it was less than twenty, I think, and more than ten, that I was invited to speak before this body. It was a novel experience, for I had never addressed a meeting of dentists before in my life. I did not even know that there was such a society as that of dentists until that night. I puzzled my brain over the question of what I could say to a body of men devoted to the dental profession, and I finally reached a certain conclusion which I expressed in the course of my speech. I was seeking for points of affinity, points of sympathetic touch, between your profession and mine. I am wiser now; I know of a good many. (Laughter.) But the conclusion I reached then, as I can recall it in part, was that both of us had a good deal to do with decayed members. (Laughter.) Another point was, that both of us were under obligations to go to the roots of things. (Laughter.) And the third point was, that we were both engaged in an effort for salvation. (Applause.)

It is always perilous to attempt to repeat a speech that one has once made. There are people I know who can do it, and it always seems right; it always fits, whether they are addressing an association of dentists or an association of undertakers, or a company of gentlemen on the Stock Exchange or a ministers' meeting, just as there are some men who have only one story, and they always tell that story, and it always seems new. I remember a man who had but one story, and that was about a gun, and he always told it; and if he could not bring it in in any other way, he would rap on the table sharply, saying, "What is that? It sounds like a gun. Oh, speaking of guns, makes me think of this," and then he would tell his story. (Laughter.)

I find great pleasure in addressing this body again. It seems to me that you have been growing younger, and I am sure you have been growing handsomer. It is quite apparent you have inverted the remark which I once heard: a gentleman looked down upon his audience, in which there were a great many bald heads, and said that it was the most extraordinary combination he ever saw, as there was the most brains and the least hair he had ever noticed together; but to-night we have a combination of hair and brains, with only here and there a pate that begins to grow shiny like mine. Those of us, however, who are in that condition may comfort ourselves with the fact that the highest degree of mental attainment is signified by the loss of one's hair.

I find great pleasure and satisfaction in addressing this body, because I have observed that in the dental profession there has been during the past twenty years a progress unequalled by that of any other profession or calling with which I am acquainted. I do not wish to assume the implied attitude of a critic by praising. It might seem that to praise you of this day and generation would be to reflect upon the past age, but that is not true, and it is always well to tell the truth. I am not disposed to flatter, but I give it as my conviction that at no time in the history of dentistry as a profession have the members of the profession stood so high in all those qualities that make up the intelligent gentleman as the profession stands to-day. It has made progress in skill; it has made progress in therapeutic and conservative ability; it has made progress and standing in the community; it has made progress in morality. To-day the profession of dentistry stands side by side with that of medicine; to-day it is a recognized branch of the medical profession, quite on a par in the scope of its ministering and in the power of its service to human kind with that of the medical profession. I suppose one must always be true to his calling, and, being a preacher, I must preach; I have a capital chance, for I do not know whether I shall ever have so many dentists to preach to again, and I am very much tempted to take this opportunity. You know the story they tell of the man who, talking to Charles Lamb, said, "Did you ever hear me preach?" "Oh," said Lamb, "I have never heard you do anything else." (Laughter.) I suppose I may plead guilty to the same charge.

In stating the subject, "The Main End," I had two things in mind. You remember the story of the preacher who always preached on eternal punishment, no matter what his text was One day, to get him off that text, it was proposed to give him a text, just as he was going to enter the pulpit, on which he should preach. He accepted the proposition, and as he entered the pulpit he was handed a sealed envelope containing a paper on which was written, "Adam, where art thou?" He began to preach, and he said, "My brethren, every man is somewhere. That is the first head. In the second place, a good many people are where they ought not to be. That is the second head. In the third place, if you don't look out a good many of you will be where you don't want to be. We will omit the consideration of the first two heads, and give our attention to the last." And then he went on and preached his usual sermon about eternal punishment. (Laughter.) But I find myself going back, not to that theme, but to one that is to me the most vital of all in the world, and which is suggested by the words of my subject.

What is life? For what are we striving? What are we seeking? There are two lines of thought that present themselves to our minds as we consider this question. What are they? The main end or aim of every man who intelligently faces life is efficiency in service, no matter what that service may be; it may be your profession, it may be mine; it may be that of any one else; but it is efficiency in things which you are called upon to do. "Failure," in big letters, is written right across the life of any man who has not clearly in mind that his first business in life is to be efficient in the thing he is called upon to do. If it is to make shoes, to make them so well that all the world will want to walk in them. If it is to practise medicine, to be so faithful and diligent in study, so broad in sympathy, so alert, so persistent in his endeavors to keep in contact with all the best knowledge that is emerging from the laboratory, that he shall be possessed of the highest efficiency which he can achieve.

There are a great many men who begin their lives with utter indifference. They have not clearly thought out what they are living for; they are going to work for money. There is many a man who begins a profession for what he can make out of it. He does it in a sordid way; he is going to make money to get on. However efficient he may be, actuated by such a motive as that, he cannot be efficient in the larger sense, for, under the influence of an ignoble motive, he will sacrifice the best elements of power which lead to the higher efficiency. Nor can it be mere success. Success is a great word with the Americans, and I don't believe they are the only devotees in the world of that ideal. A great deal has been said about the American love of the almighty dollar, but I find that the people, the world over, are after the dollar, or the mark, or the franc, or the coin of some other denomination, and it is all one thing. I was in Venice one day, and I heard one of the most extraordinary quarrels I ever listened to between two men over the purchase of half an orange which cost two-fifths of a cent. It does not make any difference whether it is cents, or centimes, or franks, or marks, or dollars, or pounds, shillings, and pence.

Success is a nobler thing than the mere material results of gain, because there is more inspiration in it, and a man who seeks success for the sake of success is a nobler man than he who seeks to do anything for what he can get out of it. No man in this world can do his best work unless he has the power to idealize his work; that is, to interpret it and see it in its largest relations. What is work? Whether it be that of the artisan or the professional man, it is the service of mankind. It is to minister to others. It is not simply the thing that he does, but it is that in its relation to the whole life. It is for the alleviation of pain that he is striving, the preservation of human power, the refinement of human life. Certainly a nation that has well-preserved and well-guarded teeth is so far a better nation, in all the qualities that go to make up a nation, than a nation which is not sound, because the teeth are related to the whole structure of the face and head, and the whole anatomical and physiological and nervous structure, and the proper care of that part is a powerful addition to the totality of the man; and the service of dentistry is, therefore, a ministering to the whole man, not only in the alleviation of pain, but in the development of that soundness of organism through which the man shall express himself most fully and effectually, and which shall be the vehicle and model, so to speak, in which shall be found the finest and noblest life.

One of the greatest thoughts that has emerged in our time to new prominence is that of the integrity of life. Man is not an ingenious production of the Creator, like a modern steamship with water-tight compartments, divided off by iron and steel, but he is one entire thing, and he needs to be right through the whole gamut of his being; and to live his best he must have a good head, a good hand, good feet, good lungs, a good heart, a good conscience, good intelligence, good imagination, good power of reasoning; and you cannot have a complete man unless each of these is fully developed. Your work and my work, and the work of every other man, rightly interpreted, relates itself to the totality of his life, and he who idealizes his work in that way enobles it and ennobles himself. The carpenter who builds a house is not merely putting together lumber, boards, and scantling; he is making a home for human lives to live and grow in; creating a place for human love and tenderness that shall flow out in the community, and every nail he drives is driven more surely because his heart goes into the stroke of his hand. If that is true of the man whom we call an artisan, it is even more true, if possible, of the man who stands on the higher plane of the professions.

So that the first thing of all is to seek efficiency in service. He is false to his profession who does not make the most of his time and opportunity to attain to the highest skill possible. He has no right to neglect those things which tell of the latest advances in dentistry, to neglect the knowledge of chemistry and other sciences which shall qualify him to be skilful. He has no right to neglect any study of the human body and its functions which will qualify him to judge of the conditions of any part of the organism with which he deals.

But that, after all, is only the beginning. The true man and true woman is working for an end, which is the fulness and completeness in life. The workman must be more than his work. If the dentist is not more than a dentist, he is not as good a dentist as he ought to be. If the farmer is not more than a farmer, he is not as good a farmer as he ought to be; and if the politician is not more than politician, he is not as good a politician as he should be. The true end of life is that culture which amplifies the whole nature, and refines and enlarges it, until man stands up a complete man before his Creator. Humboldt said, "The finest fruit earth yields up to its Maker is a finished man."

What is culture? A smattering of Greek or Latin? I suppose any of us here could chatter a few sentences in any of the ancient or modern languages; that we could "parlez vous Français," or "sprechen Sie Deutsch," but that is not culture. Is it the ability to dash off a few brilliant bars on the piano, or to play one of Chopin's studies, or one of those brilliant, incomprehensible compositions of Bach, or one of those thunder-like productions of Wagner? Is that culture? Then what is culture? It is the power to use and the disposition to approve whatever is excellent in art, in nature, in literature, in life, in action. The man who cannot see a good thing and know it from afar lacks culture. The man who can see it has the true essence of culture. But we see according to what we are, and every man brings the qualifying element which determines his vision. What you are, my friends, determines the interpretation of the world which you find, and if the world is warped and mean and low, there is something warped and low and mean in you. If you are loving and pure and aspiring, you will see the noble and the beautiful in life, and, seeing it, will grow to love it, and your whole being will enlarge.

This, then, is my message, a message which I bring with the more confidence and the more earnestness, because I know how the ideal of the professional life has developed in men who to-day are carrying on the profession of dental surgery. That is as it should be. The continual aspiration of the man or woman engaged in this noble profession to be always more than the thing they do, to be always a man, a woman, watching the mind, chastening and developing the heart, cleaning the conscience, widening the range of view, laying hold of the truth on every hand, finding out the beauty that is on every side, and bringing one's life daily more and more into harmony with the melody that rolls through all things.

So, if I may particularize for a moment, every dentist should have an avocation,—something else which he does besides the practice of his profession; something which will react upon him and widen his sympathies and develop his character; something which will make his life larger in its active operations than the one line in which he works as a professional man. I do not think it is a bad thing to have a hobby. We hear a good deal of scorn and ridicule poured out on hobby riders, but strange to say they have been the men who have pushed civilization ahead, just as some one has said it is the cranks that make the world go round. Dear old Dr. Schuyler, the man who cannot hear his own voice, and who would die of fright if he did, for it is the most terrible voice, speaking the other night of a visit he paid to an old church in England, said he found there in the floor of the church, what you often see in churches and cathedrals in England, a tomb of the dead. You will remember that Jane Austin, the writer, is buried in Winchester Cathedral, and you can stand on the stone under which she lies and read the inscription upon it. As Dr. Schuyler was going through this church he noticed a stone under his feet, the inscription on which said that it was the tomb of Jeremiah Hooper and his seven wives, who all laid under the one stone. Dr. Schuyler said to the verger who was accompanying him, "That is a good many wives, is it not?" "Well, you see," said the verger, "it were a hobby of his." (Laughter.) Now, I do not counsel you to adopt that kind of a hobby, but a hobby may be a good thing. I know a gentleman who has a lathe, and he has learned to work out the most interesting and curious and practical and valuable things on his lathe. Another man takes up something else; it may be a certain line of history which he masters, and you can go to him and he can tell you anything about it, and you can learn more from him in a few minutes than you could from anybody else, because he has put his spare time into that and he knows of it. Another man has made a study of birds. He will walk through the woods and see birds where you see none and hear notes you cannot hear. There will be a twitter in the bushes, and he will say that is such and such a bird, or you will see something flutter through the trees, and he will tell you the bird and its habits, where it goes when it migrates, and all its characteristics. His mind is full of a rich knowledge of all the birds of creation. Whatever it may be, have an avocation. Something that you will know well, that will enlarge your life and give you joy and refresh ment; for in seeking this completeness of life one needs not only an avocation, but some wider interest to link him with his fellow-men.

First of all,—and I wish I could say this to the young men who are gathering there at the college at Philadelphia, for I know something about them; I have a boy among them,—I should say to them that one of the first things, in order that a man may advance at all, is downright, persistent, unflinching sincerity. Be what you feel. We had a motto in college which read, "Not to seem, but to be," and it is a good motto for every man to take; not to stand before the community under a false disguise, not to disgrace one's profession by being one thing in his professional office and another thing when he is on the street or at his play, but to be the straightforward, sincere, true man wherever he goes.

I have a great deal of sympathy with Pat, who, being shown an epitaph on a tombstone which read, "He is not dead, but sleepeth," said, "Bedad, if I was dead I would own up to it." (Laughter.) It is a great deal better to acknowledge you are dead than to say you are sleeping. Whatever you do, stand up squarely before the whole world for the thing that you are. The great thing in life is to be. God has given us prophetic suggestions in the history of the world of what man may be. We have had such suggestions, wonderful and inexplicable, in the great Greek sculptures. The despair of the artist, the matchless grace of Praxitiles, the exquisite form of Phidias, as if God had set in the midst of the centuries a model and said, "There, that is what I

would like you to be like." The great Greek dramatist Æschylus, with his thunder note and the roll of the Ægean vibrating through his stanzas; Sophocles, with his rich melodies, and Eurypides, the Greek, who was the greatest foreshadower of Shakespeare the world ever saw; Shakespeare, the myriad-minded, as Coleridge called him, and of whom Bishop Clark said years ago, "There is but one Shakespeare, and heaven and earth were exhausted in making him." But that is not true. Heaven would show to us by these eminent examples what man can be and do, and, instead of causing us to despair, create in us the hope and endeavor and aspiration to be more than we are. And there is one man who forever induces us, forever prompts us, by the inexpressible height of his sincerity, of his truth, and his purity, not to give up or despair, but shows to what height we may aspire. Charles Lamb was in the company of his friends one evening, and they were talking about the homage due to great men. Shakespeare was mentioned, and some one then mentioned Jesus, when Lamb said, "If Shakespeare were to come into this room, we would all rise to do him homage, but if that man were to come into this room, we should all stoop to kiss the hem of his garment." He was the great physician, the great helper, who fills us not with despair, but gives hope to every man and every woman who is seeking a nobler and a higher life.

So, my friends, this is the word I would leave with you, the deep and earnest message of my heart: make the most of your magnificent profession, and make the most of it by being the biggest, the broadest, the largest and sweetest and loftiest of men, inspiring men and women, and to this end knit up your life with every interest that can broaden it, and forever be more than a mere artisan, forever be more than a mere professional worker; be men, large-minded, tall, full of sincerity and purity and truth, so that wherever you go or wherever you work, the community shall feel the uplifting power of your presence and all men shall bless your name. (Loud applause.)

*President Flanagan.*—The thanks of this Association are due and are now freely given to those who have honored us by addressing us this evening, and to all the people who have attended our meeting. The meeting will now adjourn.

(To be continued.)

